# Independent brain cortical signatures of risk for adolescent cannabis use and consequences of such use are moderated by sex

**DOI:** 10.1038/s41386-025-02249-2

**Published:** 2025-11-13

**Authors:** Jeremy J. Watts, Xavier Navarri, Patricia J. Conrod

**Affiliations:** 1 Department of Psychiatry and Addiction, University of Montreal, Montreal, QC Canada; 2 CHU Sainte-Justine Research Center, CHU Sainte-Justine, Montreal, QC Canada; 3 Department of Neuroscience, University of Montreal, Montreal, QC Canada

**Keywords:** Addiction, Ageing, Predictive markers

## Abstract

There is an accumulation of evidence linking adolescent cannabis use with variations in brain structure and function, however it remains poorly understood whether cannabis-associated variations in brain structure represent pre-existing risk factors or consequences of cannabis use. We investigated whether cannabis use propensity and within-person variations of cannabis use were associated with cortical thickness during adolescence. Adolescents (*n* = 136, 74 female) completed three neuroimaging sessions and annual assessments from 12 until 17 years of age (with 90% follow-up). Cannabis use was disaggregated into between- (vulnerability) and within-person (time-varying) components using longitudinal multi-level modelling, controlling for age, sex and alcohol use. Across the whole sample, cortical thickness was lower in years when participants’ cannabis use exceeded their own average level of cannabis use (*F*_1,25663.3_ = 3.96, *p* = 0.047; mean: −0.0023 mm/once-per-week increase). This effect was stronger in males (*F*_1,11447.7_ = 9.83, *p* = 0.0017), such that each once-per-week increase in cannabis use was associated with a 0.005 mm reduction in cortical thickness, comparable to 17.9% of the annual rate of cortical thinning (−0.028 mm/year). The strongest within-person effects of cannabis were observed in regions with the greatest expression of *CNR1*, the gene that codes for the CB1 receptor (sample: rho = −0.33, *p*_*spin*_ = .025; males: rho = −0.5, *p*_*spin*_ = .005). At the between-person level, males (but not females) also exhibited a stable cortical thickness signature associated with propensity towards cannabis use and this signature was present before cannabis exposure. These results highlight the importance of longitudinal analyses using multi-level modelling to disaggregate potential risk factors from potential consequences of substance use.

## Introduction

Cannabis is one of the most commonly used recreational drugs among North American adolescents; 25–29% of 17-year-olds (12th grade) report past-year cannabis use, with 4% reporting current daily use and 12% a history of daily use [1–3]. Whereas adolescent cannabis use has been extensively linked to increased risk of psychiatric illness, poorer academic performance and alterations of brain activity and structure [4–8], few studies have disentangled effects reflecting pre-existing risk from potential consequences of cannabis use [7, 9].

During adolescence, grey matter volume declines in the neocortex, driven by reductions in cortical thickness [10]. Lower cortical thickness has been associated with psychiatric disorders in adolescence and adulthood [11]. Despite reports of smaller orbitofrontal cortex volume in adults who use cannabis [12], these effects did not reach significance in meta-analyses involving whole brain analyses [13] or in youth who use cannabis [9].

In the largest longitudinal study reported to date (*n* = 799), initiation of cannabis use after age 14 was associated with greater age-adjusted cortical thinning in young adulthood (age 19) [14]. This study along with a complementary analysis using Bayesian causal network modelling [15] provides preliminary evidence that the effects of cannabis on cortical maturation are observed at levels of cannabis use that are common among teenagers and well below levels observed in cannabis use disorder. However, with participants cannabis-naïve at baseline and only one follow-up, the analysis relies on between-person comparisons to infer within-person effects, so it has limited ability to distinguish between processes linked to risk and those specifically linked to cannabis exposure. It is possible that faster rate of cortical thinning during adolescence contributes to risk of cannabis use while also being an observable consequence of cannabis use. It is further conceivable that distinct sets of brain regions are implicated in each type of effect. Models designed to disambiguate these effects typically require three-or-more time points and designs that separate between-person vulnerability from within-person effects (e.g. exposure) [16].

Cortical thinning is thought to be influenced by sex hormones [17], and the impact of adolescent cannabis use on brain measures and mental health consequences such as psychosis may differ by sex [18, 19]. Sex differences in the biological consequences of adolescent cannabinoid exposure have been extensively studied in rodent models [20–22]. In humans, one multi-cohort study reported that the association between cannabis and cortical thickness was greater in males [18], however that study did not investigate within-person effects.

Considering the mixed-findings of cross-sectional meta-analyses in adults and youth, and that no studies have examined maturational changes within the adolescent period related to within-person variations in cannabis use, the present study aims to examine how within-person changes in cannabis use relate to trajectories of maturation during adolescence in a longitudinal neuroimaging cohort. We hypothesise that over and above potential brain markers of risk for early onset cannabis use, within-person increases in cannabis use will be associated with greater than model-predicted changes in cortical thickness within that same year [14]. We investigate overall and sex-specific effects, and hypothesise that males will show greater within-person associations between cannabis and cortical thickness [18]. We hypothesise that a propensity toward greater cannabis use will be associated with lower-than-model-predicted cortical thickness in frontal regions reported to be predictive of future cannabis or substance use [23–26]. Finally, in line with earlier reports [14, 18], we hypothesise that the effects of within-person increases in cannabis use will be greater in brain regions with higher density of cannabinoid CB1 receptors, as estimated by *CNR1* gene expression from the Allen Human Brain Atlas [27].

## Methods

Neuroventure is a prospective cohort study composed of 151 adolescents recruited from the larger Co-Venture trial cohort [28, 29]. Briefly, participants were recruited from a normative school-based sample of 3,826 students attending the 7^th^ Grade (age 12–13) across 31 middle and high schools in the Greater Montreal Area that were randomised to deliver the personality-targeted selective prevention programme PreVenture ® (registered clinical trial number: NCT01655615). The Neuroventure sub-study was designed to investigate the developmental trajectories of adolescents with high-risk personality traits (impulsivity and sensation seeking) [29, 30]. Participants were eligible to participate if they scored one standard deviation above the school mean on sensation-seeking (*n* = 60) or impulsivity (*n* = 60) or if they scored below the mean (control group, *n* = 30) on these traits, measured using the Substance Use Risk Profile scale (SURPS) [28–30]. Each group was balanced for sex, and the same SURPS scoring cutoffs were used for both sexes. All procedures were approved by the CHU Sainte-Justine Ethics Committee. Following explanation of all study procedures, all participants provided written assent to participate, and written consent was obtained from their parent(s) or legal guardian(s) (ethics reference number: 3678).

### Demographic measures

Biological sex was self-reported. Weight and height were measured during MRI visits. Age was calculated from a participant’s date of birth to the date of the MRI visit. Socioeconomic status was assessed using the Family Affluence Scale [31].

### Substance use measures

Drug use was assessed using an adapted digital version of the Detection of Alcohol and Drug Problems in Adolescents (DEP-ADO [32]). Drug use frequency was scored to yield the number of times-per-week that an individual reported using cannabis or alcohol: ‘never’ = 0; ‘occasionally’ = 0.0625 (~3 times per year); ‘once/month’ = 0.25; ‘once-twice/week or on weekends’ = 1.5; ‘3 or more times/week but less than 7/week’ = 5; ‘every day’ = 7 times/week. Additionally, trained research staff conducted timeline follow back (TLFB) interviews with participants assessing drug use for the 180 days preceding the MRI scan, for the second and third study visits. Cannabis use assessed on the DEPADO and TLFB exhibited strong agreement (Spearman’s rho = 0.86; Supplementary Tables S1 and S2).

### Neuroimaging

Scans were acquired at the Montreal Neurological Institute (MNI) Brain Imaging Centre using a 3T Siemens Magnetom Trio scanner (visits 1 and 2), which underwent an upgrade to a Siemens Magnetom Prisma scanner at visit 3. At each visit, participants underwent T1-weighted scans using a 3D magnetisation-prepared rapid gradient echo (MPRAGE) sequence (TR = 2300 ms, TE = 2.96 ms, inversion time=900 ms, flip angle = 9 degrees, FOV phase = 93.75%, thickness = 1 mm, voxel = 1 × 1 × 1 mm, matrix dimensions = 256 × 256).

MR image quality was monitored by the MRI technician and study staff and acquisitions were repeated when scan quality was judged to be low. MRI scans were visually inspected and rated for overall quality [33]. Cortical thickness and total intracranial volume were quantified with the Freesurfer 6.0.0 longitudinal pipeline [34] using the CBrain platform [35] and then parcellated into 34 bilateral regions of interest [36]. Cross-sectional outputs were visually inspected for gross defects and processed with Qoala-T Freesurfer quality control tool with a cut-off score of 50 [37] required for inclusion in the longitudinal steps of the Freesurfer pipeline. To account for the impact of an MRI scanner upgrade from Trio (all scans for visits 1 and 2) to Prisma [38] (all scans for visit 3), we used longitudinal ComBat to harmonise cortical thickness data [39]. ComBat models included all fixed effects terms described in the primary analysis [39].

### *CNR1* gene expression

Gene expression data for donor brains (*n* = 6) and parcellated images in native space were obtained from the Allen Institute website [27] and [40], respectively. Gene expression data were processed with abagen [41] using recommendations from Arnatkevic̆iūtė et al., 2019 [40] and modifications implemented by Dear et al. [42]; (code available at https://github.com/richardajdear/AHBA_gradients). Regions were retained only if they included samples from at least three donors [42]. See Supplementary Methods for parameters set in the abagen pipeline.

### Statistical analysis

Data were analysed using a multi-level mixed model with random intercepts to be able to account for repeated measurements. The dependent variable was cortical thickness (mm). Region of interest was included in the model as a factor (34-levels). The interaction between region and number of surface holes in the uncorrected reconstruction was included in all models to control for the impact of MRI and surface reconstruction quality on regional thickness measurements [43]. Time-varying predictors were separated into between- and within-person components [16]. Between-person predictors were calculated as the average score at all time points (e.g. cannabis_average_). Within-person predictors for a given time point were calculated as the difference between the participant’s score for the individual time point and their average score across all time points (e.g. cannabis_within_).

The between-person terms were calculated as follows:

$${{{\rm{cannabis}}}}_{{{\rm{average}}}},_i=\frac{1}{{n}_{i}}{\sum }_{y=1}^{{Y}_{i}}{{Cannabis}}_{i,y}$$

where cannabis_average,*i*_ is participant *i*’s average cannabis use across the available years ${Y}_{i}$, and ${n}_{i}$ is the number of non-missing observations.

$${{cannabis}}_{i,y}={{{\rm{cannabis}}}}\; {{{\rm{use}}}}\; {{{\rm{for}}}}\; {{{\rm{participant}}}}\,i\,{{{\rm{at}}}}\; {{{\rm{year}}}}\,{y}$$

$${Y}_{i}={{{\rm{set}}}}\, {{{\rm{of}}}}\, {{{\rm{years}}}}\, {{{\rm{with}}}}\, {{{\rm{valid}}}}\, {{{\rm{data}}}}\, {{{\rm{for}}}}\, {{{\rm{participant}}}}\, {i}$$

$${n}_{i}=\left|{Y}_{i}\right|={{{\rm{number}}}}\, {{{\rm{of}}}}\, {{{\rm{study}}}}\, {{{\rm{years}}}}\, {{{\rm{with}}}}\, {{{\rm{cannabis}}}}\, {{{\rm{use}}}}\, {{{\rm{data}}}}\, {{{\rm{for}}}}\, {{{\rm{participant}}}}\,i$$

The within-person terms were calculated as follows:

$${{{\rm{cannabis}}}}_{{{{\rm{within}}}},i,T1}={{\rm{cannabis}}}_{i,T1}-{{{\rm{cannabis}}}}_{{{\rm{average}}}},_{i}$$

$${{{\rm{cannabis}}}}_{{{{\rm{within}}}},i,T2}={{\rm{cannabis}}}_{i,T2}-{{{\rm{cannabis}}}}_{{{\rm{average}}}},_{i}$$

$${{{\rm{cannabis}}}}_{{{{\rm{within}}}},i,T3}={{\rm{cannabis}}}_{i,T3}-{{{\rm{cannabis}}}}_{{{\rm{average}}}},_{i}$$

Where *T*1, *T*2, and *T*3 refer to the first, second and third MRI timepoints, respectively.

To examine specificity of cannabis-related effects, all models control for alcohol use (between- and within-person effects, calculated in the same manner as for cannabis).

We first estimated the overall annual rate of age-related cortical thinning using a model that included total intracranial volume, number of surface holes, hemisphere, sex, age, and each individual’s cortical thickness intercept (random intercept). As age-related cortical thinning has been reported to differ across brain regions, a corresponding interaction term was included (age-by-region) and if significant, was retained in the ensuing models. Although some regions of interest have been reported to follow non-linear cortical thinning trajectories, linear models may provide a good or optimal fit for most regions during adolescence [44, 45]. Inclusion of a quadratic term for age did not improve fit for the base model (supplementary Table S5), therefore quadratic terms for age were not included.

The second model expanded on this basic model by including cannabis and alcohol -within and -between variables to estimate main effects of cannabis use on cortical thickness.

A third model included interactions of cannabis terms with region of interest. This model was used to generate cannabis effect parameters for each region of interest. A fourth model built on the third model to investigate sex-by-cannabis interactions. Significant sex-by-cannabis interactions were followed-up by sex-specific models based on the cannabis main effect model and third model, which included cannabis-by-region-of-interest interactions. The second, third and fourth models controlled for alcohol use (between- and within-person variables).

To test whether a significant (between-person) cannabis use propensity phenotype preceded exposure to cannabis, we repeated the third model using cortical thickness data for timepoint 1 only, omitting the within-person cannabis and within-person alcohol use terms and restricting the sample to participants who were cannabis-naïve at timepoint 1.

To compare the similarity of regional patterns associated with within- or between-person cannabis use, we performed Spearman correlation tests on the regional estimates between the phenotypes of interest. To compare the similarity of regional patterns associated with within-person cannabis use and regional *CNR1* gene expression, we performed Spearman correlation tests.

To control for spatial autocorrelation, statistical significance of Spearman correlations was assessed using spin permutation tests [46] with 1000 rotations, carried out using the ENIGMA toolbox [47].

Sensitivity analyses were planned to control for between- and within-person effects of personality risk factors [48].

For all tests, *α* = 0.05, two-sided. Analyses were performed using lme4 [49] in R (v4.3.2).

Note about interpretation of multi-level model results: In the multi-level model framework of the present study, a within-person increase in cannabis use reflects a positive deviation from an individual’s within-person average use across all study years. For this reason, a within-person increase in cannabis use does not necessarily indicate an increase from the immediately preceding timepoint.

## Results

Sample demographics and cannabis use information are provided in Table 1. After accounting for MRI quality control and missed visits (Supplementary Table S3), the final sample included 136 participants who had at least two visits with MRI data that passed quality control and complete demographic and behavioural measures data (381 scans total). Baseline socioeconomic status for the Neuroventure sample was comparable to that of the parent cohort, which included 15% of schools in the greater Montreal area (for additional details, see online supplement to [50]).

**Table 1:** Sample characteristics and cannabis use^a^.

Characteristic	Time 1	Time 2	Time 3
Sample size, *n*^b^	132	126	123
Male/Female, *n*	61/71	55/71	55/68
Age, mean (SD), years	13.66 (0.67)	14.93 (0.44)	17.39 (0.47)
BMI, mean (SD), kg/m^2^	20.46 (3.72)	21.04 (3.56)	22.61 (3.76)
Socioeconomic status, mean (SD)	5.34 (1.59)	5.41(1.70)	5.83 (1.78)
Cannabis use frequency			
Never, *n* (%)	122 (92.4)	102 (80.9)	69 (56)
Occasional, *n* (%)	7 (5.3)	13 (10.3)	29 (23.5)
Once/month, *n* (%)	2 (1.5)	3 (2.4)	6 (4.9)
Weekends or 1–2/week, *n* (%)	0 (0)	5 (4.9)	11 (8.9)
3 or more times/week, *n* (%)	1 (0.7)	3 (2.4)	8 (6.5)

^a^For details of alcohol use, see Supplementary Table S4.

^b^Differences between sample sizes for individual timepoints and the total sample size (*n* = 136) are due to attrition at individual timepoints, for details see Supplementary Table S3.

Rates of cannabis use at various frequencies did not differ significantly between males and females (Supplementary Table S5).

### Effects of age on cortical thickness (simple model)

Overall, cortical thickness declined with age (*F*_1,25784.8_ = 2267.27, *p* < 2.2 × 10^−16^). The average annual change in cortical thickness was −0.028 mm/year (95% CI: −0.029 to −0.027 mm/year). There was also a significant age-by-region of interest interaction (*F*_33,25637.0_ = 9.49, *p* < 2.2 × 10^−16^) such that the rate of thinning varied across regions (range: −0.043 to −0.0003 mm/year).

## Full models: effects of cannabis use on cortical thickness

### Effects of within-person variation in cannabis use on cortical thickness

**Fig. 1:**
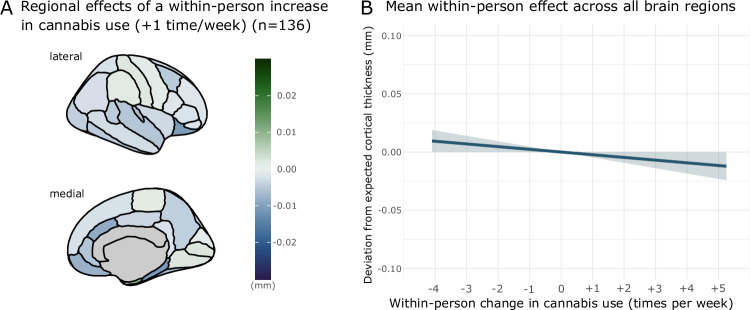
Deviations from model-predicted cortical thickness associated with within-person increases in cannabis use across the full sample. **A** regional variation in cortical thickness associated with a once-per-week within-person increase in cannabis use. **B** mean effect of increases in within-person cannabis use on cortical thickness (mean effect across all cortical regions). Expected cortical thickness refers to model-predicted cortical thickness. Model controls for image quality, intracranial volume, age, sex, region and alcohol use.

Cortical thickness was significantly associated with within-person changes in cannabis use (*F*_1,25696.8_ = 3.92, *p* = 0.048), such that an individual’s cortical thickness was lower at timepoints at which cannabis use was greater than their within-person average cannabis use across the whole study (Fig. 1A, B and Supplementary Fig. S1). The cannabis_within_*region interaction was not significant (*F*_33,25569.0_ = 0.49, *p* = 0.99). A within-person increase in cannabis use of once-per-week above a participant’s average cannabis use was associated with a reduction in cortical thickness of 2.3 × 10^−3 ^mm (95% CI: 2.1 × 10^−5^ to −0.005 mm), across all cortical regions, comparable to 8.2% of the average annual rate of cortical thinning.

### Within-person variation in cannabis use: moderating effects of sex

At the within-person level, the cannabis-by-sex interaction was significant (cannabis_within_*sex: *F*_1,25691.5_ = 6.87, *p* = 0.009). The three-way interaction of cannabis_within_*region*sex was not significant (*F*_33,25502.0_ = 0.74, *p* = 0.86). In the analyses of sex-specific effects that follow, simple main effect models and cannabis*region interaction models are reported.

#### Within-person variation in cannabis use and cortical thickness in male participants

**Fig. 2:**
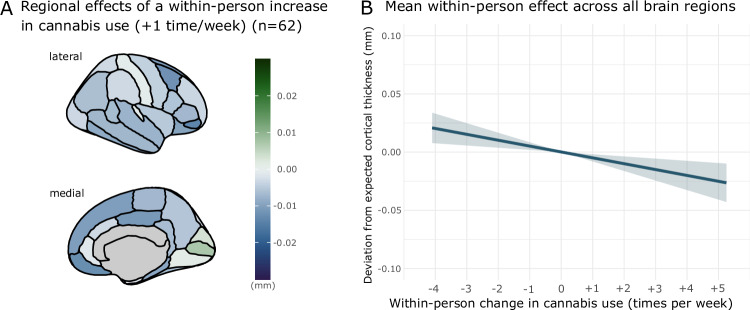
Deviations from model-predicted cortical thickness associated with within-person increases in cannabis use in male participants. **A** regional variation in cortical thickness associated with a once-per-week within-person increase in cannabis use. **B** mean effect of increases in within-person cannabis use on cortical thickness (mean effect across all cortical regions). Expected cortical thickness refers to model-predicted cortical thickness. Model controls for image quality, intracranial volume, age, region and alcohol use.

The association of within-person changes in cannabis with cortical thickness was significant in male participants (*F*_1,11447.7_ = 9.83, *p* = 0.0017; Fig. 2), such that cortical thickness was lower in years that a participant reported greater-than-their own average cannabis use (Fig. 2A, B). This within-person association did not differ significantly by region of interest (cannabis_within_*region: *F*_33,11362.9_ = 0.29, *p* = 1.0; Fig. 2A, B and Supplementary Fig. S2). A within-person increase in cannabis use of once-per-week above a participant’s average cannabis use was associated with a 0.005 mm (95% CI: −0.008 to −0.002 mm) reduction in cortical thickness, across all cortical regions, comparable to 17.9% of the annual rate of cortical thinning.

#### Within-person variation in cannabis use and cortical thickness in female participants

The association of within-person changes in cannabis use on cortical thickness was not significant in female participants (*F*_1,14113.4_ = 0.26, *p* = .61) and did not differ significantly across brain regions (cannabis_within_*region: *F*_33,14003.1_ = 0.98, *p* = 0.50; Supplementary Fig. S3).

### Within-person effects and spatial distribution of *CNR1* mRNA

**Fig. 3:**
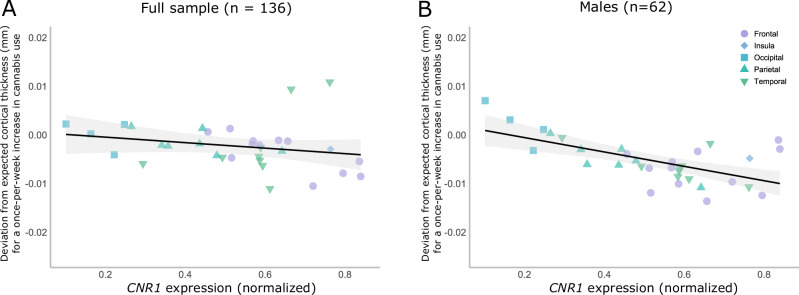
Spatial correlations of regional *CNR1* gene expression with regional estimates for the effect of within-person escalation in cannabis use. Association between regional *CNR1* gene expression and regional effects of within-person escalation of cannabis use on cortical thickness in (**A**) the full sample (*n* = 136), and **B** males only (*n* = 62). Expected cortical thickness refers to model-predicted cortical thickness. Models control for image quality, intracranial volume, age, region and alcohol use.

The strength of the within-person effect of cannabis use on cortical thickness was significantly associated with *CNR1* gene expression (Fig. 3), such that regions with greater *CNR1* expression exhibited stronger within-person cannabis effects across the full sample (rho = −0.33, *p*_*spin*_ = 0.025; Fig. 3A) and in male participants (rho = −0.5, *p*_*spin*_ = 0.005; Fig. 3B).

### Cannabis use propensity (between-person) and cortical thickness

Across the whole sample, cortical thickness was not significantly associated with cannabis use at the between-person level (*F*_1,130.7_ = 0.659, *p* = 0.42), accounting for age, sex, brain region and alcohol use. There was a significant cannabis_average_*region interaction (*F*_33,25569.0_ = 3.54, *p* = 3.05 × 10^−11^) indicating that adolescents prone to more frequent cannabis use exhibit a specific neural signature on cortical thickness.

### Cannabis use propensity: moderating effects of sex

At the between-person level, the cannabis-by-sex interaction was significant (cannabis_average_*sex: *F*_1,129.7_ = 5.13, *p* = 0.025). Further, the three-way cannabis_average_*region*sex interaction was significant (*F*_33,25503.0_ = 2.83, *p* = 1.19 × 10^−07^), indicating that the patterns of regional cortical thickness associated with proneness to more frequent cannabis use differed between males and females. In the analyses of sex-specific effects that follow, simple main effect models and cannabis*region interaction models are reported.

#### Cannabis use propensity and cortical thickness in males

**Fig. 4:**
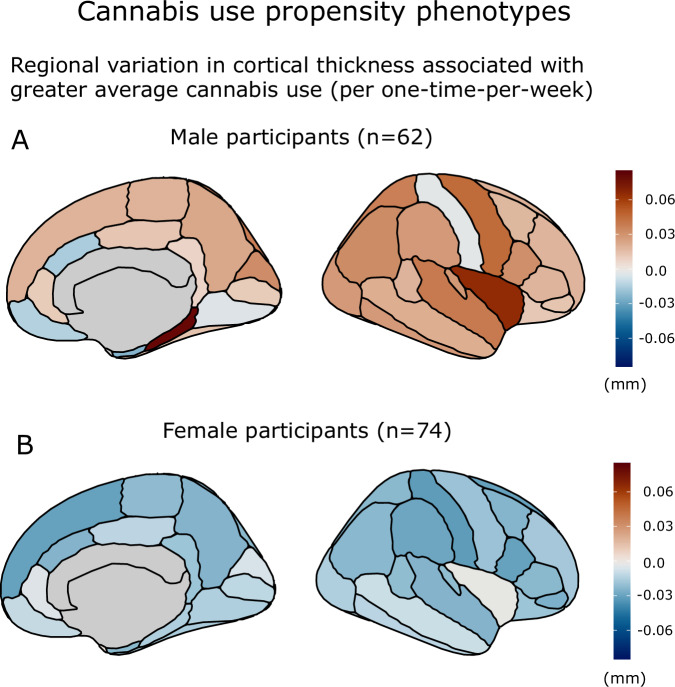
Regional effect estimates for cannabis use propensity phenotypes in male and female participants. Regional variation in cortical thickness associated with greater average cannabis use (effect scaled to depict the effect of greater cannabis use of once-per-week) in (**A**) male participants, and **B** female participants. Models control for image quality, intracranial volume, age, region and alcohol use.

Cannabis use propensity (average cannabis use throughout adolescence) was not significantly associated with overall cortical thickness in male participants (*F*_1,57.8_ = 1.77, *p* = 0.18). A significant interaction indicated that cannabis use propensity was associated with region-specific patterns of deviations from model-predicted cortical thickness (cannabis_average_*region: *F*_33,11362.9_ = 4.45, *p* = 3.297 × 10^−16^; Fig. 4A and Supplementary Fig. S4).

#### Cannabis use propensity and cortical thickness in females

Cannabis use propensity was not significantly associated with overall cortical thickness in females (*F*_1,70.0_ = 3.31, *p* = 0.073). However, a similar interaction indicated that the association of cannabis use propensity with cortical thickness differed significantly across brain regions (cannabis_average_*region: *F*_33,14003.1_ = 4.45, *p* = 3.297 × 10^−16^; Fig. 4B and Supplementary Fig. S5).

#### Sensitivity analysis: cannabis propensity phenotype at baseline

##### Males

At the first timepoint in cannabis-naïve male participants (*n* = 56), cannabis use propensity was not significantly associated with cortical thickness overall (*F*_1,56_ = 2.96, *p* = 0.09) but differed significantly across brain regions (cannabis_average_*region: *F*_33,3585.2_ = 1.72, *p* = .0065). The cortical signature at baseline, when participants were cannabis-naïve, was very similar to the cortical signature yielded from the between-person by region effect of cannabis reported in the main analysis (spatial correlation: rho = 0.94, *p*_*spin*_ < 2.2 × 10^−16^).

##### Females

At the first timepoint in cannabis-naïve female participants (*n* = 66), cannabis use propensity was not significantly associated with cortical thickness overall (*F*_1,66_ = 1.90, *p* = 0.17). Further, the association of cannabis use propensity with cortical thickness did not differ significantly across regions of interest (cannabis_average_*region: *F*_33,4255.2_ = 0.79, *p* = 0.79).

### Correlation between cannabis use propensity phenotypes in males and females

The cortical thickness signatures associated with cannabis use propensity in males and females were not significantly correlated, whether testing the phenotype across all three timepoints (rho = 0.17, *p*_*spin*_ = 0.21) or at the first timepoint alone (rho = 0.14, *p*_*spin*_ = 0.23).

### Cannabis use propensity phenotypes and spatial distribution of *CNR1* mRNA

The distribution of *CNR1* mRNA was not significantly associated with patterns of cortical thickness variations associated with propensity toward more frequent cannabis use in male (rho = −0.17, *p*_*spin*_ = 0.28) or female participants (rho = 0.18, *p*_*spin*_ = 0.28).

### Personality traits

The results of primary analyses were not meaningfully altered when controlling for impulsivity or sensation seeking at the between- and within-person levels (Supplementary materials).

## Discussion

To our knowledge, this study is the first to investigate the impact of within-person changes in cannabis use on adolescent brain development using a model with more than two timepoints and that can disambiguate a brain signature associated with vulnerability from one that is potentially linked to drug harms.

Within-person increases in cannabis use were associated with lower-than-predicted cortical thickness. At timepoints when participants’ cannabis use exceeded their own average level of use, cortical thickness was lower than predicted, considering participants’ age, sex and use of alcohol and individual cortical thickness intercept. The effect suggests that escalation of cannabis use is associated with cortical thinning over-and-above predicted age-related cortical thinning and over-and-above regional and group differences in cortical thickness linked to risk for cannabis use. Existing reports from single-follow-up designs relied on between-person effects to infer a dose-dependent relationship between cannabis use and cortical thinning but are consistent with the findings nevertheless [14].

The present findings are consistent with a growing body of evidence supporting a causal relationship between adolescent cannabis use and maturation of the cerebral cortex in humans [14, 18]. The spatial association of the strength of the within-person effect and *CNR1* expression is in agreement with other reports from large adolescent cohorts [14, 18]. Further, the association of within-person variation in cannabis use was specific to cortical thickness, as similar results were not observed for cortical surface area (Supplementary Results). The present study provides evidence that in the *same individual*, initiation or escalation of cannabis use is associated with greater cortical thinning, extending our understanding of cannabis use and brain maturation.

The current findings also suggest that this effect differed across sexes such that the within-person effect was more pronounced in male participants. Cannabis use frequency did not differ significantly by sex in this sample (Supplementary Table S5). In males, an increase of one time per week of cannabis use was associated with cortical thinning equivalent to ~17.9% of the annual rate of age-related cortical thinning. The present results would predict that males who escalate their cannabis use during adolescence would be the most affected.

An independent longitudinal study of adolescents did not observe significant sex differences in the association between cannabis use and cortical thickness [14]. However, compared to the present analysis, participants in this earlier study were older at baseline and follow-up, had fewer follow-up MRI scans (one vs. two), and a longer interval between MRI visits (5 vs. 2 years) [14]. Only the present study employed a multi-level design that permitted simultaneous modelling of associations of brain structure with cannabis use propensity as well as time-varying changes in cannabis use. This unique design revealed distinct brain signatures associated with risk for and consequences of adolescent cannabis use. The former showed region specificity, and the latter demonstrated a brain-wide effect on cortical thickness.

At the between-person level, greater average cannabis use was associated with a regionally varying phenotype that was sex specific. In males only, this phenotype preceded exposure to cannabis, in line with a vulnerability phenotype associated with propensity towards greater cannabis use. This cannabis-by-region interaction was driven by divergence in the direction of cannabis-thickness associations, with greater cannabis use associated with a tendency toward thinner cortex in some frontal regions (e.g. caudal anterior cingulate cortex, medial orbitofrontal cortex) but toward thicker-than-predicted cortex in others (Fig. 4). The association of greater propensity for cannabis use with a tendency for lower cortical thickness in the orbitofrontal and anterior cingulate cortices, in contrast to most other regions, is in line with reports that reduced thickness or volume of these structures predict early substance use initiation [23–26, 51]. In contrast to results at the within-person level, greater average cannabis use was associated with sex-specific and regionally varying phenotypes both for cortical thickness and surface area (Supplementary results), in line with a vulnerability phenotype given that surface area is thought to be largely determined at earlier stages of development [52, 53]. Future studies are needed to interrogate the specificity of this vulnerability phenotype with respect to substance use patterns and whether it extends to related behaviours such as problematic internet use or pathological gambling [54–56].

There are numerous reports of sex differences in cannabis use risk factors, cannabis use behaviours and the effects of cannabis, complicating the task of understanding sex-related effects in observational studies [57–62]. For instance, an earlier study reported that males were more likely to report using cannabis at higher frequencies, quantities and were more likely to use high-potency forms of cannabis products [59]. Controlled studies provide evidence that females are more sensitive to cannabis, requiring lower doses than males to achieve comparable subjective effects [60–62]. Finally, although the vulnerability phenotype in males resembles that from other reports for future substance use and related behaviours [54–56], future analyses incorporating a broader range of risky behaviours could provide insight into sex differences in observed in the present study. Thus, although males and females in our study were matched for personality risk factors and reported comparable cannabis use frequency, sex differences in behaviour [57] and/or in cannabis product type, dosage, or potency [59–62] may have contributed to the observed sex differences and should be investigated further.

Preclinical studies have reported sex-differences in the biological and behavioural consequences of adolescent THC exposure [20–22], however such differences remain poorly understood in humans [63]. Cortical thinning during adolescence is related to sex hormones and pubertal timing [17, 64], thus longitudinal cohort studies that follow adolescents from a younger age and include detailed measurement of pubertal stage and sex hormones could provide insight into sex differences in the association of cannabis use with brain maturation in humans.

The present work has several methodological strengths. To our knowledge, The present study achieved the highest retention rate to date among longitudinal neuroimaging studies of similar or greater size that investigated cannabis use and cortical thickness. In contrast, a larger cohort (*n* = 2223) included only 36% of their baseline sample in their 5-year longitudinal analyses of adolescent users (*n* = 799) [14]. Cortical thickness was quantified using a longitudinal image analysis pipeline optimised for detecting within-person changes in brain structure [34]. Finally, the levels of cannabis use observed in this study are reflective of those commonly observed in adolescents [3], whereas most other longitudinal studies of cannabis use and brain structure have focused on near-daily users or youth with cannabis use disorder (CUD) [9] (with the exception of the cohort study described previously [14]). Our findings further support a link between cannabis use and greater cortical thinning at levels of use frequently observed in adolescents, and potentially below those typical of CUD [65–67].

This work should be interpreted with a mind to several considerations. Although toxicology of biological matrices could complement self-report data, hair samples require at least moderate levels of use, hair and urine have a limited temporal window for detection and cannot provide detailed information about substance-use frequency [68]. Moreover, self-reported cannabis use was assessed annually and was consistent across questionnaire and interview methods (Timeline Follow-back, Supplementary Table S1 [69]). The study did not collect information about type or amount of cannabis used, route of administration, or Δ^9^-THC content [70, 71]. This analysis did not include detailed assessment of pubertal stage, which could have improved modelling of age-related changes in the brain or substance exposure effects sensitive to pubertal stage [17, 64].

The present study was embedded within an intervention trial that led to reductions in substance use disorder [50]. The association of within-person variation in cannabis use and cortical thickness is expected to be independent of intervention, and such an analysis was beyond the scope of the present work. Further, the stable between-person phenotype observed in males was present at baseline, before individuals received an intervention. Adolescent cannabis use is associated with increased risk for development of psychiatric disorders (e.g. [5]), thus further work is needed to understand the relationship between brain structure and vulnerability to development of substance use or psychiatric disorders. Results of this analysis were not meaningfully altered when controlling for the between- and within-person effects of personality risk factors on cortical thickness (Supplementary results), further dissociating risk from potential drug use effects. Male and female participants were recruited according to the same criteria for sensation-seeking and impulsivity traits. Therefore, interpreting differences in results for males and females in this context robustly controls for sex-related differences in these traits.

Although larger sample sizes are often preferred for investigating relationships between brain and behaviour or environment, many of the concerns that apply to cross-sectional univariate analyses are mitigated by design features of the present study [72]. Longitudinal design and within-person modelling improve power and replicability [73]. The Neuroventure sample was enriched for youth with high scores on impulsivity or sensation seeking, but included those without elevated scores, enabling analysis of substance use in a focused developmental sample while maintaining generalisability [72, 73]. Reliability of behavioural measures was strengthened by repeated measurements during five annual assessments [69, 74]. Finally, by explicitly modelling between- and within-person effects, the present analysis avoided conflating those effects which, as reported in this work, exhibited distinct relationships to cortical thickness (magnitude, direction, spatial pattern, etc.) [73].

## Conclusion

The present study provides evidence relating within-person increases in cannabis use to brain-wide reductions in cortical thickness that are more pronounced in male adolescents. According to the results of this study, adolescent male cannabis users exhibited a brain vulnerability phenotype present before exposure to cannabis and greater brain vulnerability to cannabis use. Studies designed to quantify exposures should be prioritised to better understand these sex differences. Nevertheless, these findings support ‘Lower-Risk Cannabis Use Guidelines’ recommendation to delay cannabis use until after adolescence [75]. Distinct within- and between-person effects of cannabis use were observed across three time points during adolescence. These findings highlight the importance of longitudinal designs and analyses that disaggregate brain-related predictors of cannabis use from its potential consequences.

## Supplementary information

Supplementary material

## Data Availability

The data supporting the findings of this study are not publicly available due to the terms of the assent and consent forms. The data supporting the findings of this study are available upon reasonable request from Dr. Patricia Conrod.
