# Supplementary material for: Independent brain cortical signatures of risk for adolescent cannabis use and consequences of such use are moderated by sex
Source: Neuropsychopharmacology. 2025 Nov 13;51(2):497–505. doi: 10.1038/s41386-025-02249-2 (PMC12708861; doi:10.1038/s41386-025-02249-2)
Supplement: Supplementary file 1 — Supplementary material [file 41386_2025_2249_MOESM1_ESM.pdf]

Supplementary material for:

Independent brain cortical signatures of risk for adolescent cannabis use and consequences of such use are moderated by sex

Jeremy J Watts, PhD[1,2], Xavier Navarri, MSc[1,3], Patricia J. Conrod, PhD[1,2]

Affiliations

1. Department of Psychiatry and Addiction, University of Montreal, Montreal, Canada
2. CHU Sainte-Justine Research Centre, CHU Sainte-Justine, Montreal, Canada.
3. Department of Neuroscience, University of Montreal, Montreal, Canada

Supplementary Methods

- Participants
- Self-report measures of substance use
    - Supplementary table S1 Agreement between TLFB and DEPADO (Pearson)
    - Supplementary table S2 Agreement between TLFB and DEPADO (Spearman)
- MRI image processing
- *CNR1* gene expression
- Figure generation
- Details of excluded or absent MRI scans at each time point
    - Supplementary table S3. Reasons for absence or exclusion of MRI scans at each time point

Supplementary Results

- Additional drug use information
    - Supplementary table S4. Alcohol use in male and female participants
    - Supplementary table S5. Cannabis use in male and female participants
- Model comparisons with quadratic terms for age
    - Supplementary table S6. Model comparisons with quadratic term for age
- Supplementary table S7. Normalized expression of CNR1
- Supplementary sensitivity analyses
    - Controlling for Personality Traits
        - Effects of within-person variation in cannabis use on cortical thickness: initiation or escalation of cannabis use
        - Cannabis use propensity (between-person) and cortical thickness
    - Comparison of regional cortical phenotypes:
    - Effects of within-person variation in cannabis use on cortical surface area
    - Cannabis use propensity (between-person) and cortical surface area
    - Cannabis use propensity and cortical surface area: moderating effects of sex
        - Cannabis use propensity and surface area in males
        - Cannabis use propensity and surface area in females

Supplementary Figures

Effects of within-person variation in cannabis use

- **Supplementary Figure S1**. Regional deviations in cortical thickness associated with within-person increases in cannabis use in all participants (n=136).
- **Supplementary Figure S2**. Regional deviations in cortical thickness associated with within-person increases in cannabis use in male participants (n=62).
- **Supplementary Figure S3**. Regional deviations in cortical thickness associated with within-person increases in cannabis use in female participants (n=74).

Effects of between-person differences in cannabis use throughout adolescence

- **Supplementary Figure S4**. Regional deviations in cortical thickness associated with greater average cannabis use in male participants (n=62).
- **Supplementary Figure S5**. Regional deviations in cortical thickness associated with greater average cannabis use in female participants (n=74).

Supplementary References

Supplementary methods:

Participants

Exclusion criteria: major neurodevelopmental disorders (e.g., autism), uncorrectable visual impairment, hearing deficits, uninterruptible central nervous system medication, any contraindications (e.g., pregnancy) for MRI (Bourque, Baker et al. 2016). Half of the high-risk youth were exposed to a brief personality-targeted cognitive behavioural intervention in the 7th grade (Bourque, Baker et al. 2016, O'Leary-Barrett, Mâsse et al. 2017).

Self-report measures of substance use

DEPADO (Landry, Tremblay et al. 2004) measures of substance use frequency were available for all participants included in the present study. At time 2, timeline follow back (TLFB; (Sobell and Sobell 1992)) data for 23 participants were not available. At time 3, TLFB data were not available for 4 participants. Individuals with missing TLFB data were removed from this analysis. DEPADO categories of substance use frequency were converted into 'times per week' as described in the main manuscript. Supplementary tables S1 and S2 present results of parametric and non-parametric correlations between frequency of use as measured by the DEPADO and TLFB.

Supplementary table S1. Agreement between TLFB and DEPADO (Pearson)

		r	p
Time 2	Cannabis	0.86	$<2.2\times10^{-16}$
	Alcohol	0.45	2.29×10^{-6}
Time 3	Cannabis	0.86	$<2.2\times10^{-16}$
	Alcohol	0.75	$<2.2\times10^{-16}$

Supplementary table S2. Agreement between TLFB and DEPADO (Spearman)

		rho	p
Time 2	Cannabis	0.77	$<2.2\times10^{-16}$
	Alcohol	0.72	$<2.2\times10^{-16}$
Time 3	Cannabis	0.80	$<2.2\times10^{-16}$
	Alcohol	0.71	$<2.2\times10^{-16}$

MRI image processing

Freesurfer 6.0.0: Briefly, this pipeline includes B1 bias field correction, skull stripping, segmentation of grey and white matter, talairach transformation, cortical surface reconstruction, cortical parcellation and estimation of grey matter metrics including cortical thickness and surface area (Fischl 2012). Cross-sectional data were then passed on to the Freesurfer longitudinal pipeline, which improves reliability for within-person data (Reuter, Schmansky et al. 2012).

Freesurfer processing was conducted on a compute cluster provided by the Digital Research Alliance of Canada and the Freesurfer 6.0.0 tools on CBrain (Sherif, Rioux et al. 2014).

Qoala-T: Qoala-T is a quality control tool for Freesufer-processed data that was originally validated with a developmental sample including adolescents (Klapwijk, Van De Kamp et al. 2019). Qoala-T uses supervised-learning to generate quality-control ratings and scans scoring below 50 were not included in the analysis (Klapwijk, Van De Kamp et al. 2019).

CNR1 gene expression

Regional microarray expression data were obtained from 6 post-mortem brains (1 female, ages 24.0--57.0, 42.50 +/- 13.38) provided by the Allen Human Brain Atlas (AHBA, https://human.brain-map.org; (Hawrylycz, Lein et al. 2012)). Data were processed with the abagen toolbox (version 0.1.3; https://github.com/rmarkello/abagen) using a 69-region volumetric atlas (Desikan, Ségonne et al. 2006), independently aligned to each donor's native MRI space.

The report of processing of AHBA data was generated as an automated output of the abagen pipeline before being reviewed by authors with minor revisions for manuscript preparation. All generated text was released under a creative commons CC0 license.

Before processing, AHBA samples were filtered to include only those from cortical regions (Dear, Wagstyl et al. 2024).

First, microarray probes were reannotated using data provided by (Arnatkevičiūtė, Fulcher and Fornito 2019); probes not matched to a valid Entrez ID were discarded. Next, probes were filtered based on their expression intensity relative to background noise (Quackenbush 2002), such that probes with intensity less than the background in >=50.00% of samples across donors were discarded , yielding 32,669 probes. When multiple probes indexed the expression of the same gene, we selected and used the probe with the most consistent pattern of regional variation across donors (i.e., differential stability; (Hawrylycz, Miller et al. 2015), calculated with:

$$\Delta_S(p) = \frac{1}{\binom{N}{2}} \sum_{i=1}^{N-1} \sum_{j=i+1}^{N} \rho\left(B_i(p), B_j(p)\right)$$

Where, ρ is Spearman's rank correlation of the expression of a single probe, p, across regions in two donor brains B_i and B_j and N is the total number of donors. Here, regions correspond to the structural designations provided in the ontology from the AHBA.

To increase spatial coverage, tissue samples in the right hemisphere were mirrored into the left hemisphere (Romero-Garcia, Whitaker et al. 2018). Samples were assigned to brain regions in the provided atlas if their native voxel coordinates were within 2 mm of a given parcel. To reduce the potential for misassignment, sample-to-region matching was constrained by hemisphere and gross structural divisions (i.e., cortex, subcortex/brainstem, and cerebellum, such that e.g., a sample in the left cortex could only be assigned to an atlas parcel in the left cortex; (Arnatkevičiūtė, Fulcher and Fornito 2019)). All tissue samples not assigned to a brain region in the provided atlas were discarded.

Inter-subject variation was addressed by normalizing tissue sample expression values across genes using a robust sigmoid function (Fulcher, Little and Jones 2013):

$$x_{\text{norm}} = \frac{1}{1 + \exp\left(-\frac{x - \langle x \rangle}{\text{IQR}_x}\right)}$$

where $\langle x \rangle$ is the median and IQR_x is the normalized interquartile range of the expression of a single tissue sample across genes. Normalized expression values were then rescaled to the unit interval:

$$x_{\text{scaled}} = \frac{x_{\text{norm}} - \min(x_{\text{norm}})}{\max(x_{\text{norm}}) - \min(x_{\text{norm}})}$$

Gene expression values were then normalized across tissue samples using an identical procedure. Samples assigned to the same brain region were averaged separately for each donor, yielding a regional expression matrix for each donor with 69 rows, corresponding to brain regions, and 15,946 columns, corresponding to the retained genes. Finally, 33 of 69 regions remained after filtering for regions with ≥ 3 donors.

Figures

Brain map and linear effect figures were created using ggseg (Mowinckel and Vidal-Piñeiro 2020) and visreg (Breheny and Burchett 2017) in R (v4.3.2). Where necessary, minor changes to figure formatting were made using Inkscape (https://inkscape.org/).

Details of excluded or absent MRI scans at each time point

Supplementary table S3. Reasons for absence or exclusion of MRI scans at each time point

	Time 1	Time 2	Time 3
Missed visit	0	3	0
No scan due to orthodontic braces	0	5	5
Withdrew from study	0	10	15
Substance use measures unavailable	1	0	0
Incidental finding	2	2	2
Epilepsy	1	1	1
[a]MRI visual quality control fail	2	2	2
Failed QoalaT	0	2	3
Failed Freesurfer processing	1	0	0
Only 1 scan passing QC	12	0	0

[a]MRI quality was inspected at the time of scan. If the first T1-weighted image was judged to be of low quality, the sequence was repeated up to twice during the same MRI visit. Only the highest quality MRI for a given MRI visit was considered for the present analysis.

Supplementary results:

Substance use by sex

Supplementary table S4. Alcohol use in male and female participants

Characteristic	Time 1		Time 2		Time 3	
	n		n		n	
Sample size[a]	132		126		123	
	male	female	male	female	male	female
n	61	71	55	71	55	68
Cannabis use frequency						
Fisher's exact test	p = .002		p = .83		p = .47	
Never (%)	20 (33)	36 (50)	12 (22)	20 (28)	6 (11)	7 (10)
Occasional (%)	41 (67)	28 (39)	31 (56)	37 (52)	19 (35)	24 (35)
Once/month (%)	0 (0)	3 (8)	7 (13)	6 (8)	11 (20)	20 (29)
Weekends or 1-2/week (%)	0 (0)	4 (6)	5 (9)	7 (10)	17 (31)	16 (24)
3 or more times/week (%)	0 (0)	0 (0)	0 (0)	1 (1)	2 (4)	1 (1)

[a]Differences between sample sizes for individual timepoints and the total sample size (n=136) are due to participant attrition at individual timepoints, for details see Supplementary Table S3.

Supplementary table S5. Cannabis use in male and female participants

Characteristic	Time 1		Time 2		Time 3	
	n		n		n	
Sample size[a]	132		126		123	
	male	female	male	female	male	female
n	61	71	55	71	55	68
Cannabis use frequency						
Fisher's exact test	p = .92		p = .22		p = .50	
Never (%)	56 (92)	66 (93)	48 (87)	54 (76)	32 (58)	37 (54)
Occasional (%)	4 (7)	3 (4)	3 (5)	10 (14)	11 (20)	18 (26)
Once/month (%)	1 (2)	1 (1)	0 (0)	3 (4)	4 (7)	2 (3)
Weekends or 1-2/week (%)	0 (0)	0 (0)	3 (5)	2 (3)	4 (7)	7 (10)
3 or more times/week (%)	0 (0)	1 (1)	1 (2)	2 (3)	4 (7)	4 (6)

[a]Differences between sample sizes for individual timepoints and the total sample size (n=136) are due to participant attrition at individual timepoints, for details see Supplementary Table S3.

Model comparisons with and without quadratic terms for age

Inclusion of age^2 and/or age^2*region terms did not lead to meaningful improvement in model fit compared to the base model (likelihood ratio test; supplementary table S5).

**Base model**	**Including Age^2**	**Including Age^2*region**
`ThickAvg_combat ~`	`ThickAvg_combat ~`	`ThickAvg_combat ~`
`LR * region +`	`LR * region +`	`LR * region +`
`holes_LR * region +`	`holes_LR * region +`	`holes_LR * region +`
`Age * region +`	`Age * region +`	`Age * region +`
	`Age_sq +`	`Age_sq * region +`
`SEX + ICV_z + (1 | CODE)`	`SEX + ICV_z + (1 | CODE)`	`SEX + ICV_z + (1 | CODE)`

```
Outcome:
ThickAvg_combat Cortical thickness

Predictors:
    LR: hemisphere (two levels: left, right)

    holes_LR: total number of surface holes in uncorrected reconstruction in left or right
              hemisphere

    Age: participant age at timepoint

    Age_sq: square of participant age at timepoint

    region: brain region (34 regions × 2 hemispheres)

    SEX: participant sex (two levels: left, right)

    ICV_z: standardized intracranial volume

Random effects:
    (1 | CODE): Random intercept for participant
```

Supplementary table S6. Model comparisons with quadratic terms for age

	# params	AIC	BIC	logLik	deviance	df	p (χ^2)
Age<br>Age*region	140	-28595	-27452	14437	-28875		
Age, Age*region<br>Age^2	141	-28593	-27442	14437	-28875	1	.88
Age, Age*region<br>Age^2, Age^2*region	174	-28534	-27113	14441	-28882	34	1.0

Model tests reflect contrasts against the base model (Age, Age*region)

***CNR1* expression by region**

Supplementary table S7 Normalized expression of *CNR1*

region	*CNR1* (Normalized expression)	RANK
caudal anterior cingulate	0.839	1
rostral anterior cingulate	0.836	2
medial orbitofrontal	0.795	3
insula	0.764	4
temporal pole	0.762	5
lateral orbitofrontal	0.72	6
entorhinal	0.665	7
pars orbitalis	0.657	8
posterior cingulate	0.642	9
rostral middle frontal	0.634	10
parahippocampal	0.612	11
fusiform	0.594	12
middle temporal	0.589	13
superior temporal	0.587	14
superior frontal	0.586	15
inferior temporal	0.584	16
pars opercularis	0.57	17
pars triangularis	0.568	18
caudal middle frontal	0.516	19
paracentral	0.512	20
bankssts	0.493	21
precuneus	0.479	22
precentral	0.457	23
supramarginal	0.443	24
isthmus cingulate	0.436	25
inferior parietal	0.357	26
superior parietal	0.341	27
transverse temporal	0.294	28
postcentral	0.265	29
lingual	0.248	30
lateral occipital	0.222	31
cuneus	0.164	32
pericalcarine	0.102	33
frontal pole	NA	NA

Supplementary sensitivity analyses

Controlling for Personality Traits

Effects of within-person variation in cannabis use on cortical thickness: initiation or escalation of cannabis use

Across all participants, the association of within-person changes in cannabis use and cortical thickness was not meaningfully different when controlling for SURPS impulsivity score ($F_{1,25663.0}$=3.84, p=.05), or sensation seeking score ($F_{1,25629.2}$=33.67, p=.055).

Males: The association of within-person changes in cannabis use and cortical thickness for male participants was significant when controlling for SURPS impulsivity score ($F_{1,11379.3}$=8.87, p=.0029), or sensation seeking score ($F_{1,11380.2}$=10.41, p=001).

Females: The association of within-person changes in cannabis use and cortical thickness for female participants was not significant when controlling for SURPS impulsivity score ($F_{1,14047.5}$=0.79, p=0.37), or sensation seeking score ($F_{1,14046.1}$=0.59, p=.44).

Cannabis use propensity (between-person) and cortical thickness

Males: The interaction between average cannabis use and cortical thickness was significant when controlling for impulsivity ($F_{33,11361.9}$=4.46, p=3.21×10^{-16}) or sensation-seeking ($F_{33,11361.9}$=4.46, p=3.13×10^{-16}).

Females: The interaction between average cannabis use and cortical thickness was significant when controlling for impulsivity ($F_{33,14002.1}$=1.85, p=0.002) or sensation-seeking ($F_{33,14002.1}$=1.86, p=0.002).

Comparison of regional cortical phenotypes:

Spatial similarity of cortical thickness signatures for cannabis use propensity and within-person cannabis use in male participants

The regional pattern of the cannabis propensity phenotype (figure 4A and supplementary figure S3) was not significantly correlated with the regional pattern for within-person increases in cannabis use (figure 2 and supplementary figure S2; rho=-.26, p=.14).

Effects of within-person variation in cannabis use on cortical surface area:

Cortical surface area was not significantly associated with within-person variation in cannabis use across all regions ($cannabis_{within}$: $F_{1,25682.3}=0.21$, $p=.65$). The $cannabis_{within}$*region interaction was not significant ($F_{33,25568.3}=0.27$, $p=.99$). The three-way $cannabis_{within}$*region*sex interaction was not significant ($F_{33,25501.4}=0.60$, $p=.97$).

Cannabis use propensity (between-person) and cortical surface area

Across the whole sample, surface area was not significantly associated with cannabis use at the between-person level ($F_{1,129.6}=0.12$, $p=.73$), accounting for age, sex, brain region, alcohol use, and intracranial volume. There was a significant $cannabis_{avg}$*region interaction ($F_{33,25568.3}=3.45$, $p=8.04\times10^{-11}$) indicating that adolescents prone to more frequent cannabis use exhibit a specific neural signature on surface area. The three-way $cannabis_{within}$*region*sex interaction was significant.

Cannabis use propensity and cortical surface area: moderating effects of sex

The three-way $cannabis_{between}$*region*sex interaction was significant ($F_{33,25535.5}=7.59$, $p<2.2\times10^{-16}$), indicating that the patterns of regional surface area associated with proneness to more frequent cannabis use differed between males and females.

Cannabis use propensity and surface area in males

Cannabis use propensity (average cannabis use throughout adolescence) was not significantly associated with overall surface area in male participants ($F_{1,57.6}=0.10$, $p=.76$). A significant interaction indicated that cannabis use propensity was associated with region-specific patterns of deviations from model-predicted surface area ($cannabis_{between}$*region: $F_{33,11395.8}=9.43$, $p<2.2\times10^{-16}$).

Cannabis use propensity and surface area in females

Cannabis use propensity (average cannabis use throughout adolescence) was not significantly associated with overall surface area in female participants ($F_{1,69.3}=0.05$, $p=.82$). A significant interaction indicated that cannabis use propensity was associated with region-specific patterns of deviations from model-predicted surface area ($cannabis_{between}$*region: $F_{33,14035.7}=2.94$, $p=3.60\times10^{-8}$).

Supplementary Figures

Effects of within-person variation in cannabis use: initiation or escalation of cannabis use

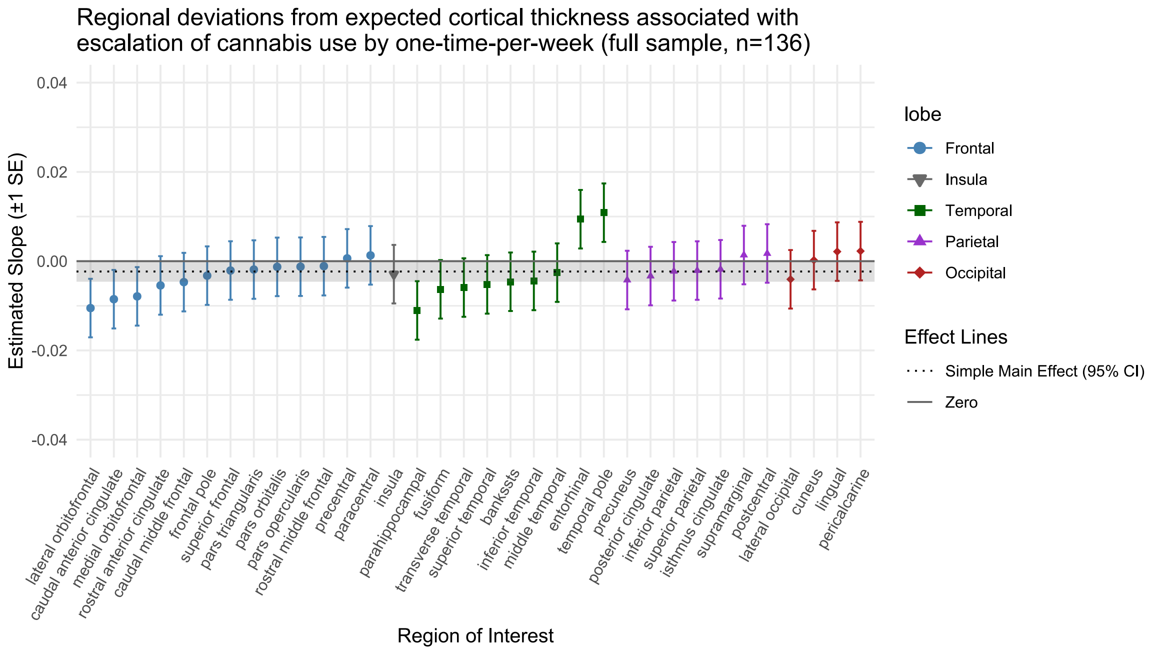

Supplementary Figure S1. Regional deviations in cortical thickness associated with within-person increases in cannabis use in all participants (n=136). The simple main effect is depicted as a dotted line surrounded by shaded area (95% confidence intervals) across the width of the figure. Estimated slope reflects the deviation from expected cortical thickness for each within-person increase in cannabis use of one-time-per-week. Linear mixed models were fit using a shared error term across regions of interest. Model controls for image quality, intracranial volume, sex, age, region, and alcohol use

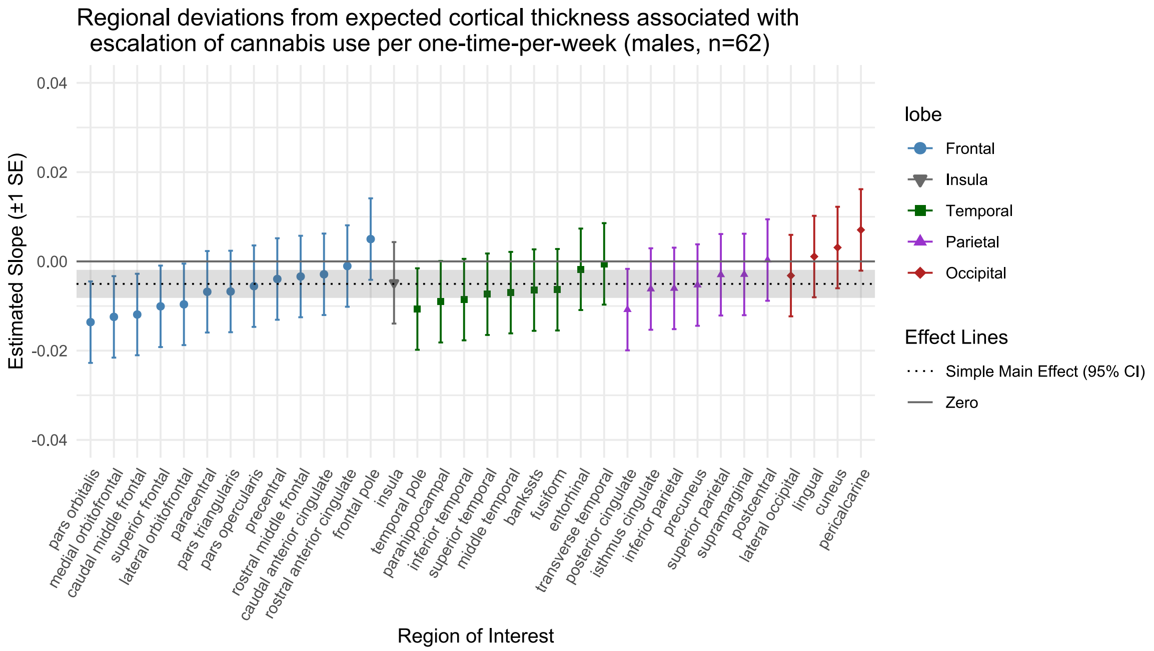

Supplementary Figure S2. Regional deviations in cortical thickness associated with within-person increases in cannabis use in male participants (n=62). Estimated slope reflects the deviation from expected cortical thickness for each within-person increase in cannabis use of one-time-per-week. Linear mixed models were fit using a shared error term across regions of interest. Model controls for image quality, intracranial volume, age, region, and alcohol use.

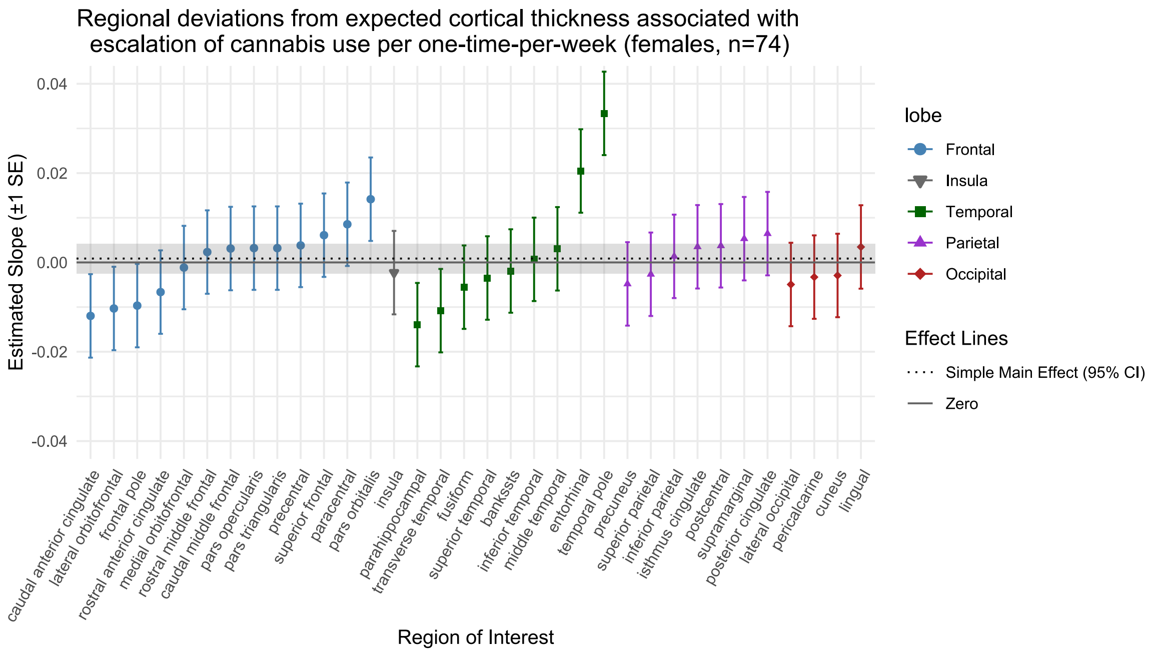

Supplementary Figure S3. Regional deviations in cortical thickness associated with within-person increases in cannabis use in female participants (n=74). Estimated slope reflects the deviation from expected cortical thickness for each within-person increase in cannabis use of one-time-per-week. Linear mixed models were fit using a shared error term across regions of interest. Model controls for image quality, intracranial volume, age, region, and alcohol use.

Effects of between-person differences in cannabis use throughout adolescence: cannabis use propensity

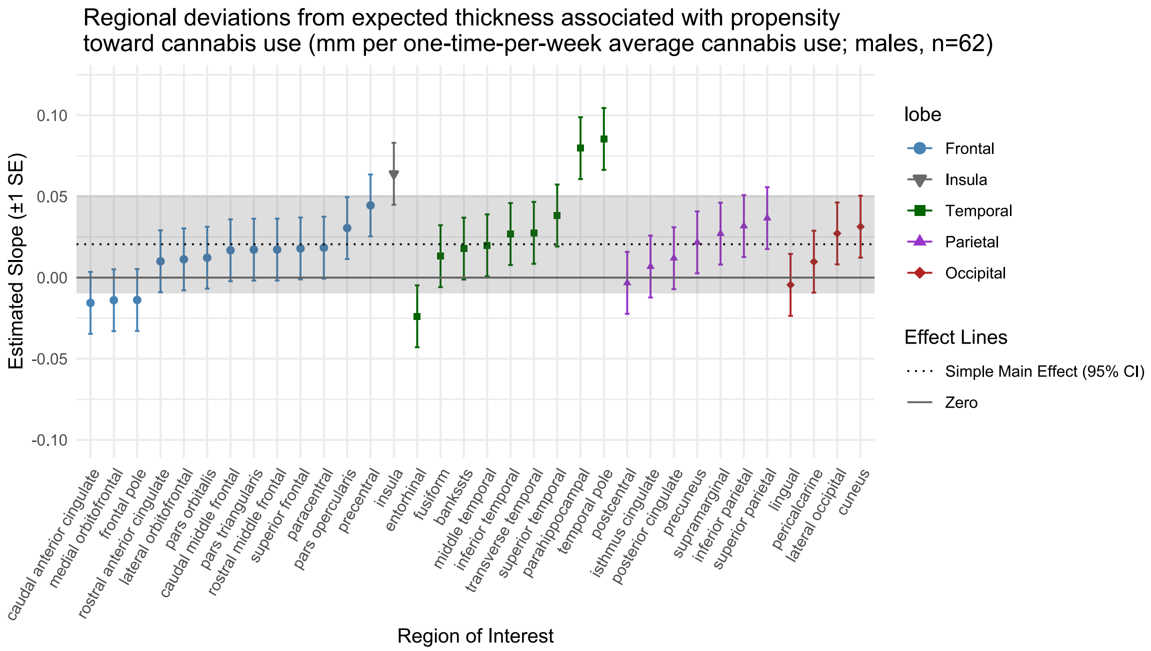

Supplementary Figure S4. Regional deviations in cortical thickness associated with greater average cannabis use in male participants (n=62). Estimated slope reflects the deviation from expected cortical thickness for each increase in average cannabis use of one-time-per-week. Models were fit using a shared error term across regions of interest. Model controls for image quality, intracranial volume, age, region, and alcohol use

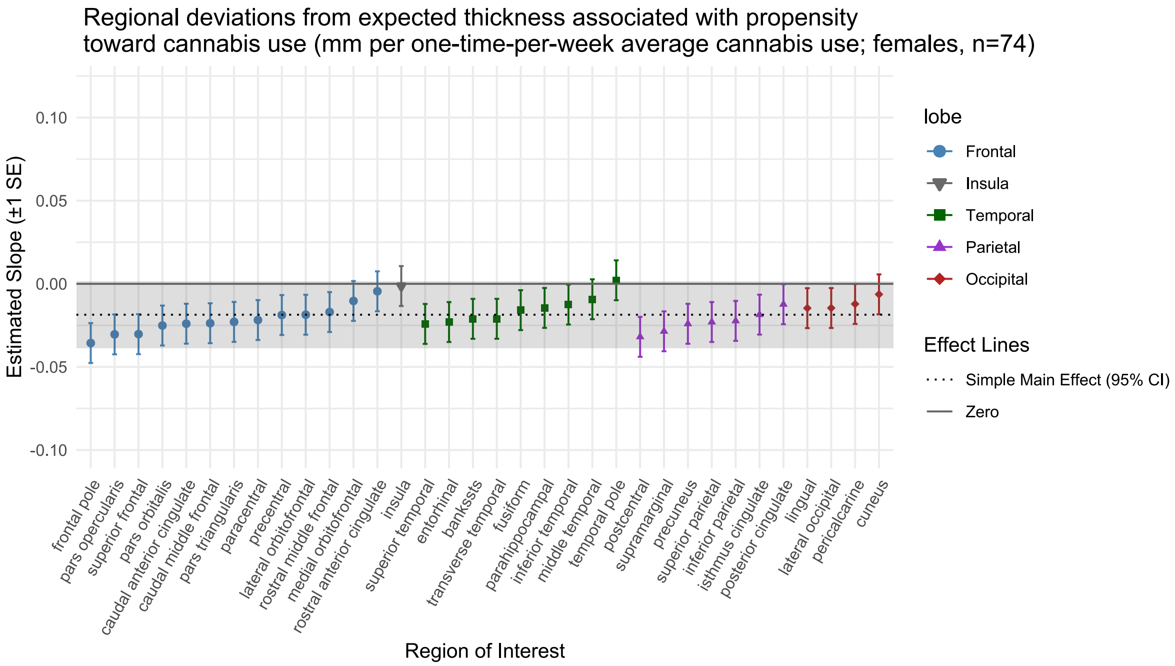

Supplementary Figure S5. Regional deviations in cortical thickness associated with greater average cannabis use in female participants (n=74). Estimated slope reflects the deviation from expected cortical thickness for each increase in average cannabis use of one-time-per-week. Models were fit using a shared error term across regions of interest. Model controls for image quality, intracranial volume, age, region, and alcohol use

Supplementary References

Arnatkevičiūtė, A., B. D. Fulcher and A. Fornito (2019). "A practical guide to linking brain-wide gene expression and neuroimaging data." Neuroimage **189**: 353-367.

Bourque, J., T. E. Baker, A. Dagher, A. C. Evans, H. Garavan, M. Leyton, J. R. Séguin, R. Pihl and P. J. Conrod (2016). "Effects of delaying binge drinking on adolescent brain development: a longitudinal neuroimaging study." BMC psychiatry **16**(1): 1-9.

Breheny, P. and W. Burchett (2017). "Visualization of regression models using visreg."

Dear, R., K. Wagstyl, J. Seidlitz, R. D. Markello, A. Arnatkevičiūtė, K. M. Anderson, R. A. Bethlehem, L. B. C. Consortium, A. Raznahan and E. T. Bullmore (2024). "Cortical gene expression architecture links healthy neurodevelopment to the imaging, transcriptomics and genetics of autism and schizophrenia." Nature Neuroscience **27**(6): 1075-1086.

Desikan, R. S., F. Ségonne, B. Fischl, B. T. Quinn, B. C. Dickerson, D. Blacker, R. L. Buckner, A. M. Dale, R. P. Maguire and B. T. Hyman (2006). "An automated labeling system for subdividing the human cerebral cortex on MRI scans into gyral based regions of interest." Neuroimage **31**(3): 968-980.

Fischl, B. (2012). "FreeSurfer." Neuroimage **62**(2): 774-781.

Fulcher, B. D., M. A. Little and N. S. Jones (2013). "Highly comparative time-series analysis: the empirical structure of time series and their methods." Journal of the Royal Society Interface **10**(83): 20130048.

Hawrylycz, M., J. A. Miller, V. Menon, D. Feng, T. Dolbeare, A. L. Guillozet-Bongaarts, A. G. Jegga, B. J. Aronow, C.-K. Lee and A. Bernard (2015). "Canonical genetic signatures of the adult human brain." Nature neuroscience **18**(12): 1832-1844.

Hawrylycz, M. J., E. S. Lein, A. L. Guillozet-Bongaarts, E. H. Shen, L. Ng, J. A. Miller, L. N. Van De Lagemaat, K. A. Smith, A. Ebbert and Z. L. Riley (2012). "An anatomically comprehensive atlas of the adult human brain transcriptome." Nature **489**(7416): 391-399.

Klapwijk, E. T., F. Van De Kamp, M. Van Der Meulen, S. Peters and L. M. Wierenga (2019). "Qoala-T: A supervised-learning tool for quality control of FreeSurfer segmented MRI data." Neuroimage **189**: 116-129.

Landry, M., J. Tremblay, L. Guyon, J. Bergeron and N. Brunelle (2004). "La Grille de dépistage de la consommation problématique d'alcool et de drogues chez les adolescents et les adolescentes (DEP-ADO): développement et qualités psychométriques." Drogues, santé et société **3**(1): 20-37.

Mowinckel, A. M. and D. Vidal-Piñeiro (2020). "Visualization of brain statistics with R packages ggseg and ggseg3d." Advances in Methods and Practices in Psychological Science **3**(4): 466-483.

O'Leary-Barrett, M., B. Mâsse, R. O. Pihl, S. H. Stewart, J. R. Séguin and P. J. Conrod (2017). "A cluster-randomized controlled trial evaluating the effects of delaying onset of adolescent substance abuse on cognitive development and addiction following a selective, personality-targeted intervention programme: the Co-Venture trial." Addiction **112**(10): 1871-1881.

Quackenbush, J. (2002). "Microarray data normalization and transformation." Nature genetics **32**(4): 496-501.

Reuter, M., N. J. Schmansky, H. D. Rosas and B. Fischl (2012). "Within-subject template estimation for unbiased longitudinal image analysis." Neuroimage **61**(4): 1402-1418.

Romero-Garcia, R., K. J. Whitaker, F. Váša, J. Seidlitz, M. Shinn, P. Fonagy, R. J. Dolan, P. B. Jones, I. M. Goodyer and E. T. Bullmore (2018). "Structural covariance networks are coupled to expression of genes enriched in supragranular layers of the human cortex." Neuroimage **171**: 256-267.

Sherif, T., P. Rioux, M.-E. Rousseau, N. Kassis, N. Beck, R. Adalat, S. Das, T. Glatard and A. C. Evans (2014). "CBRAIN: a web-based, distributed computing platform for collaborative neuroimaging research." Frontiers in neuroinformatics **8**: 54.

Sobell, L. C. and M. B. Sobell (1992). Timeline follow-back: A technique for assessing self-reported alcohol consumption. Measuring alcohol consumption: Psychosocial and biochemical methods, Springer**:** 41-72.
